# Wireless Networks under a Backoff Attack: A Game Theoretical Perspective

**DOI:** 10.3390/s18020404

**Published:** 2018-01-30

**Authors:** Juan Parras, Santiago Zazo

**Affiliations:** Information Processing and Telecommunications Center, Universidad Politécnica de Madrid, ETSI Telecomunicación, Av. Complutense 30, 28040 Madrid, Spain; santiago.zazo@upm.es

**Keywords:** CSMA/CA, backoff attack, game theory, Nash equilibrium, correlated equilibrium, regret matching

## Abstract

We study a wireless sensor network using CSMA/CA in the MAC layer under a backoff attack: some of the sensors of the network are malicious and deviate from the defined contention mechanism. We use Bianchi’s network model to study the impact of the malicious sensors on the total network throughput, showing that it causes the throughput to be unfairly distributed among sensors. We model this conflict using game theory tools, where each sensor is a player. We obtain analytical solutions and propose an algorithm, based on Regret Matching, to learn the equilibrium of the game with an arbitrary number of players. Our approach is validated via simulations, showing that our theoretical predictions adjust to reality.

## 1. Introduction

The remarkable advances and proliferation in wireless networks in recent years have brought a significant interest in the security and threats to these networks. Specially wireless sensor networks can be the target of many different attacks due to the limited capabilities of the sensors, as some recent surveys show (for instance, [1,2]). A very important kind of attack addressed to these networks is the backoff attack. It affects to the Medium Access Control (MAC) layer when a CSMA/CA (carrier-sense medium access with collision avoidance) scheme is used to regulate the access to the medium. The backoff mechanism minimizes the risk of collision, i.e., that two or more stations transmit simultaneously, by deferring transmissions during a certain random time period: the backoff window. In a backoff attack, a sensor uses a lower backoff window than the rest of the sensors, thus obtaining a higher throughput at expense of the other sensors [3].

Backoff attacks are a real threat to wireless sensor networks. Firstly, because network adapters are highly programmable [4], thus allowing sensors to modify their backoff parameters. In addition, secondly, because many MAC layer protocols proposed for wireless sensor networks make use of CSMA as medium access mechanism, for instance, SMAC [5], WiseMAC [6], TMAC [7] and DSMAC [8]. Two surveys on MAC layer protocols [9,10] show that CSMA is the most common access mechanism in contention based MAC protocols.

Some studies that treat backoff attacks, such as [11,12], focus only in the defense mechanism. However, any attack is a conflict between the attacker agents and the defense mechanism. In order to better model this conflict, we will make use of game theory tools: a branch of mathematics used to model conflict. This approach is pretty popular: [13] is a survey on game theory approaches to multiple access situations in wireless networks and [14] is another survey focused on CSMA methods.

Two works which study backoff attacks in wireless networks are [4,15]. We differ from these works in the following points and contributions:

- We assume that the defense mechanism lies in a sensor, to which other sensors communicate. We model the conflict individually between each communicating sensor—which can behave normally or attack —and the defending sensor. This is the case of networks with a star topology, in which a central sensor receives the packets of the rest of the network. This topology appears, for instance, in hierarchical routing protocols [16]: in these protocols, the sensors are clustered in order to be energy efficient (one recent example is [17]), each cluster following a star topology. Yet our approach could be adapted to other network topologies (e.g., to a mesh); however, we focus in star topology in this work for simplicity. By differentiating between attacking sensors and the defense sensor, we use a heterogeneous network model: the attacking sensors are greedy and want the maximum individual throughput they can obtain, whereas the defending sensor tries to divide fairly the total throughput among the sensors that communicate with it. This makes our model different from [4,15]: each sensor may have different interests, which is a more complex and realistic situation.
- We use Bianchi’s model to estimate the total network throughput and use this metric as game payoff: we try to enforce a fair use of the network total throughput. By modeling the total throughput, we contribute to provide a deeper insight on how different parameters influence the fairness of the network. Namely, we will show that fairness is related to the backoff parameters and the number of greedy sensors.
- We solve our game both analytically and empirically, proposing a simple algorithm to obtain the game solution, based on regret-matching (RM) algorithm [18]. Our contribution here is twofold: on one side, we provide a theoretical framework to the backoff attack problem, and solve it analytically. On the other side, we provide a simple algorithm that learns the solution to the game, which is very simple to implement, even in sensors with low computational capabilities. This makes our model both well theoretically founded and also, practical to implement in real-life situations.

Finally, note that we refer to the sensors as stations if we study the network from the MAC protocol perspective or as players or agents if we are studying the network from the game theoretic perspective.

The rest of the work goes as follows. In Section 2, we explain the CSMA/CA mechanism as used in the IEEE 802.11 standard [19]. We study this case because there is no standard for MAC layer protocols in wireless sensor networks and the 802.11 defines a very well known CSMA/CA implementation. The results we obtain can be applied to other MAC access mechanisms based on CSMA/CA. Then, in Section 3 we will use Bianchi model to obtain the theoretical throughput of the network and show how greedy stations do have a strong impact on the network throughput. In Section 4, we model the CSMA/CA problem using game theory tools, and solve it in Section 5. After, in Section 6, we will simulate the solutions proposed and analyze the results. Finally, in Section 7 we will draw some conclusions.

## 2. CSMA/CA in IEEE 802.11

The IEEE 802.11 standard [19] defines the MAC and physical (PHY) layer specifications for a wireless local area network (WLAN). Each device connected using this standard is known as station (STA). The access to the shared medium can be regulated using the Distributed Coordination Function (DCF), which uses CSMA/CA to access the medium.

The basic mechanism used by the DCF in IEEE 802.11 standard is CSMA/CA to control the medium access and a positive acknowledgment frame (ACK): if no ACK is received, there is a retransmission. CSMA/CA operates using two procedures: a carrier sense (CS) which determines whether the channel is busy (i.e., other station is transmitting) or idle (i.e., no other station is transmitting); and a backoff procedure which determines when a station should start transmitting.

A station willing to transmit invokes the CS mechanism to determine whether the channel is idle or not. If it is busy, the station defers the transmission until the channel is idle without interruption for a fixed period of time. After, the station starts a counter, called backoff, for an additional deferral time before transmitting: the station transmits when its backoff counter reaches 0. This procedure minimizes collisions among multiple stations that have been deferring to the same event. The backoff follows a uniform random variable in the interval [0,CW−1], where CW stands for contention window. If a collision is detected when a station transmits, its CW is duplicated (binary exponential backoff) and the backoff procedure starts over. When the station has transmitted the packet, it waits for an ACK; if none is received in a certain time (ACK timeout), the station starts the transmission procedure again. This mechanism is known as Basic Access (BA), and is based on a two-way handshaking.

The standard also defines an alternative procedure, based on a four-way handshaking, called request-to-send/clear-to-send (RTS/CTS). In this case, the transmitter station sends a RTS frame to the receiver, using the BA mechanism described above. The RTS frame is used to reserve the medium: when the receiver station receives a RTS, proceeds to reserve the channel for some time, sending a CTS frame to indicate that the channel reservation was successful. When the transmitting receives the CTS frame, starts transmitting its packet; when it finishes, if the transmission was successful, the receiving STA sends a positive ACK. While the channel is reserved, the rest of stations remain silent. The RTS/CTS procedure helps easing the hidden node problem [20,21] and provides a higher throughput than the BA mechanism when the MAC payload is large [22].

## 3. Network Throughput under Backoff Modification

### 3.1. Theoretical Network Throughput

The 802.11 standard does not provide a way to estimate the network throughput that is achieved. The best-known model to estimate the throughput in a network is Bianchi’s model [22], which provides expressions both for BA and RTS/CTS mechanisms. The main advantage of this model is that it provides analytical expressions to determine the network throughput. It assumes saturation of the network, that is, that each station always has a packet to transmit. This assumption could be relaxed using more complex models (as [23]).

The CSMA/CA mechanism described in Section 2 assumes that all stations will respect the backoff procedure. However, the stations can modify their backoff in such a way that they can obtain a higher throughput, at expense of other stations [4,15]. In order to analyze these effects, we will use Bianchi’s model [22] to estimate the total network throughput. The results will be used in the posterior sections to study how to enforce network throughput fairness. This model relies on the computation of the following system for each of the *i* stations of the network:

(1) $$\left\{\begin{array}{c} \tau_{i}=\frac{2}{1+W_{i}+p_{i}W_{i}\sum_{j=0}^{m_{i}-1}{\left(2p_{i}\right)}^{j}}\\ p_{i}=1-\prod_{j\neq i}\left(1-\tau_{j}\right) \end{array}\right.$$

where $p_{i}$ is the collision probability for station *i* (the probability that station *i* observes a collision while transmitting a packet, which Bianchi’s model assumes to be constant) and $\tau_{i}$ is the probability that station *i* transmits a packet. The system (1) assumes a binary exponential backoff, where the contention window CW lies in the interval $\left[W,CW_{max}\right]$, where *m* is the maximum backoff stage, defined as $CW_{max}=2^{m}W$ where *W* is the minimum size of the contention window.

Let us assume that we have a network with *n* stations, split into two different classes. There are $n_{1}$ stations characterized by using a binary exponential backoff as described by IEEE 802.11 standard, and thus, following (1). Also, there are $n_{2}=n-n_{1}$ stations using a uniformly distributed backoff in the range $\left[0,W_{2}-1\right]$, whose expression [22] is:

(2) $$\tau_{i}=\frac{2}{1+W_{i}}$$

The probabilities $\tau_{i}$ and $p_{i}$ are the same for all the members of each class. Hence, (1) becomes:

(3) $$\left\{\begin{array}{c} \tau_{1}=\frac{2}{1+W_{1}+p_{1}W_{1}\sum_{j=0}^{m_{1}-1}{\left(2p_{1}\right)}^{j}}\\ \tau_{2}=\frac{2}{1+W_{2}}\\ p_{1}=1-{\left(1-\tau_{1}\right)}^{n_{1}-1}{\left(1-\tau_{2}\right)}^{n_{2}}\\ p_{2}=1-{\left(1-\tau_{1}\right)}^{n_{1}}{\left(1-\tau_{2}\right)}^{n_{2}-1} \end{array}\right.$$

where the subscript *i* denotes the class of a station. Now, we will obtain the total throughput of the network [22,23]. The probability that there is at least one station transmitting is denoted as $P_{tr}$:

(4) $$P_{tr}=1-\prod_{i=1}^{n}\left(1-\tau_{i}\right)=1-{\left(1-\tau_{1}\right)}^{n_{1}}{\left(1-\tau_{2}\right)}^{n_{2}}$$

and hence, $1-P_{tr}$ is the probability that no station is transmitting. The probability that there is exactly one station of class *i* transmitting, $P_{s,i}$, is:

(5) $$\left\{\begin{array}{c} P_{s,1}=\tau_{1}{\left(1-\tau_{1}\right)}^{n_{1}-1}{\left(1-\tau_{2}\right)}^{n_{2}}\\ P_{s,2}=\tau_{2}{\left(1-\tau_{1}\right)}^{n_{1}}{\left(1-\tau_{2}\right)}^{n_{2}-1} \end{array}\right.$$

and the probability that there are two or more stations transmitting simultaneously (i.e., the collision probability), denoted by $P_{c}$, is obtained as the total probability minus the probabilities of having exactly none or one station transmitting:

(6) $$P_{c}=1-\sum_{i}P_{s}-\left(1-P_{tr}\right)=P_{tr}-n_{1}P_{s,1}-n_{2}P_{s,2}$$

Now, we obtain the expected duration of a slot time, $T_{slot}$. We define $T_{s}$ as the time to count down a backoff unit (i.e., the time that lies between two consecutive calls to the CS method when the channel was sensed idle), $T_{t}$ as the time duration of a successful transmission and $T_{c}$ as the time duration of a collision. We assume that the stations of both classes share the same duration of a successful transmission and the same duration of a collision. Thus, $T_{slot}$ is:

(7) $$T_{slot}=\left(1-P_{tr}\right)T_{s}+\left(n_{1}P_{s,1}+n_{2}P_{s,2}\right)T_{t}+P_{c}T_{c}$$

We consider $T_{p}$ the payload information time duration in a successful transmission and we assume that all stations share the same $T_{p}$. We define $S_{i}$, the throughput ratio for station *i*, as the fraction of time used by station *i* to successfully transmit payload bits. $S_{i}$ is obtained as:

(8) $$S_{i}=\frac{P_{s,i}T_{p}}{T_{slot}}=\frac{P_{s,i}T_{p}}{\left(1-P_{tr}\right)T_{s}+\left(n_{1}P_{s,1}+n_{2}P_{s,2}\right)T_{t}+P_{c}T_{c}}$$

In (8), we could use units of time for the magnitudes $T_{p}$, $T_{s}$, $T_{t}$ and $T_{c}$, or measure its length in bits, as long as the units are the same for the four parameters. Finally, the total network throughput, defined as the fraction of the time spent by all the stations transmitting successfully payload bits, is:

(9) $$S=\sum_{i=1}^{N}S_{i}=n_{1}S_{1}+n_{2}S_{2}$$

The parameters $T_{s}$, $T_{t}$ and $T_{c}$ are obtained from the 802.11 standard. $T_{s}$ is the empty slot time. In case of using BA mechanism, we have [22]:

(10) $$\left\{\begin{array}{c} T_{c}^{ba}=H+T_{p}+DIFS+\delta \\ T_{t}^{ba}=H+T_{p}+SIFS+\delta +ACK+DIFS+\delta \end{array}\right.$$

where *H* is the total header transmission time (adding PHY and MAC layers headers), DIFS and SIFS are interframe spacing defined in the standard, ACK is the transmission time of an ACK and δ is the propagation delay. We also consider that all payloads have the same size, whose transmission time is $T_{p}$. In case of using RTS/CTS mechanism, we have [22]:

(11) $$\left\{\begin{array}{c} T_{c}^{rts}=RTS+DIFS+\delta \\ T_{t}^{rts}=RTS+SIFS+\delta +CTS+SIFS+\delta +T_{t}^{ba} \end{array}\right.$$

Comparing (10) and (11), we see that BA mechanism uses less time for a successful transmission, whereas the time spent in a collision depends on the payload size. Intuitively, in case of large payloads and a high collision probability, RTS/CTS could achieve a higher throughput, since less time is spent on retransmissions and that might compensate the longer time spent on transmitting. Indeed, this is observed in [22].

### 3.2. Simulation 1: Network Throughput and Fairness

Now, we will make use of the expressions derived in the previous section to analyze the impact of having $n_{2}$ stations that follow a uniform backoff, and hence, do not respect the binary backoff procedure. The values used for time durations are the same as in [22], extracted from 802.11 standard, and can be seen in Table 1. Observe that we consider two different payload lengths, a short one, $T_{p,s}$, and a long one, $T_{p,l}$. We consider that the stations of class 1 follow the IEEE 802.11 standard binary backoff mechanism (normal stations), with $W_{1}=CW_{min,1}=32$, $CW_{max,1}=1024$ and hence, $m_{1}=5$. The stations of class 2 (malicious stations) will follow a uniformly distributed backoff in the interval $\left[0,W_{2}-1\right]$.

**Figure 1:**
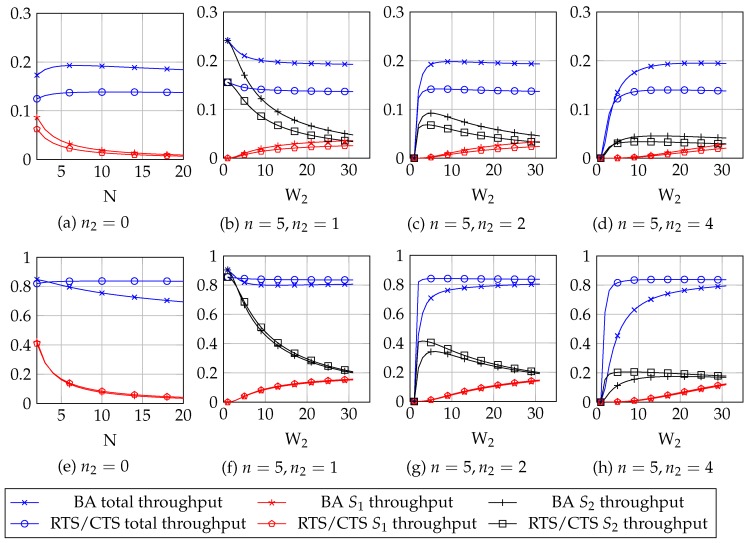
Throughput (*S*) results for the simulations using Bianchi’s model with short payload, $T_{p,l}$, (**a**–**d**) and long payload, $T_{p,l}$, (**e**–**h**). In cases (**a**) and (**e**), there are no malicious stations; in cases (**b**–d) and (**f**–**h**) there are malicious stations. $S_{1}$ is the throughput of normal stations, $S_{2}$ the throughput of malicious stations.

With these values, we obtain the throughput for each station using (8) and (9) for these cases:

- Using the large payload: $T_{p}=T_{p,l}$. We test four cases: first, we consider that $n_{2}=0$, that is, all stations follow the binary exponential backoff and we vary the number of stations for n∈[1,20]. Then, we fix the number of stations to n=5 and simulate for $n_{2}\in \left\{1,2,4\right\}$, that is, for respectively 1, 2 and 4 malicious stations. We show the results for different values of $W_{2}$, namely, for $W_{2}\in \left[1,W_{1}\right]$. The obtained results are in Figure 1.
- Using the short payload, $T_{p}=T_{p,s}$. We test the same four cases than we did for the large payload case. The obtained results are in Figure 1.

### 3.3. Discussion

The results showed in Figure 1 show that:

- The throughput of normal stations decreases significantly for low values of $W_{2}$. This is independent of the number of malicious stations, the mechanism used (BA or RTS/CTS) and the payload size. This happens because the malicious stations use lower backoffs and hence, they have higher chances to win the contention procedure against normal stations. This causes that the throughput is not fairly distributed among stations. As $W_{2}$ increases (i.e., the malicious stations behave more similarly to the normal ones) the throughputs difference becomes smaller.
- If there is only one malicious station, this station consumes the major part of the network throughput for low $W_{2}$, because it usually wins the contentions. This is independent of the mechanism used (BA or RTS/CTS) and the payload size. Yet when there are more than one malicious stations, the total throughput becomes 0 for $W_{2}=1$, because there are some stations trying to access the network that will always collide. As the $W_{2}$ value increases, we observe that the throughput for the malicious stations also increases, presenting a maximum value which depends on the total number of stations in the network and the $W_{2}$ parameter. Also, as $n_{2}$ increases, the throughput a malicious station obtains decreases: it is better for a malicious station to be the only malicious station in the network.
- RTS/CTS mechanism provides higher throughput when using larger payloads: in Figure 1e–h, RTS/CTS curves are always above BA curves. The opposite happens when using short payloads.

Hence, if in a network using CSMA/CA there is one or more stations which can modify the binary exponential backoff procedure used by 802.11, the throughput that each station gets can be seriously affected. This happens using both BA or RTS/CTS mechanisms. The results obtained in this section show that network fairness is seriously affected by a backoff attack; the next sections will propose a solution to this situation using game theory tools.

## 4. Problem Modelling as a Static Game

### 4.1. Introduction to Static Games

We define a static game as follows [24]:

**Definition 1** (Static game)**.** *A static game G is a triple $\langle N_{p},A,u\rangle$, where:*

- $N_{p}$ is the number of players, numbered as $1,\ldots ,N_{p}$.
- A is the set of actions available to all players. The pure actions available to player i are denoted by $a_{i}$, with $a_{i}\in A_{i}$, being $A_{i}$ the set of actions available to player i. A is defined as $A\equiv \prod_{i}A_{i}$. A is assumed to be a compact (i.e., bounded and closed) subset of $R^{N_{p}}$.
- *u is a continuous function that gives the game payoffs:*
  (12) $$u:\prod_{i}A_{i}\to R^{N_{p}}$$

For our game, the players are the sensors and the actions are to attack or not to attack in case of greedy sensors, and detect a malicious behavior or not for the sensor that is receiving the packets. We will use discrete sets of actions (i.e., $A_{i}$ are finite sets). Thus, the payoff functions will be discrete. Each of this discrete actions will be denoted as pure actions. Also, if there are $N_{p}=2$ players, the payoff functions for each player can be expressed using a matrix $R_{i}$. The matrices will have dimension m×n, where *m* is the number of actions for player 1 and *n* the number of actions for player 2. Hence, the element $r_{a_{1},a_{2}}$ of the matrix $R_{i}$ corresponds to the payoff for player *i* when player 1 plays pure action $a_{1}$ and player 2 pure action $a_{2}$ [24].

A game is said to be a zero-sum game if the sum of the payoffs of all players equals zero, that is, $\sum_{i}u_{i}\left(a\right)=0,\forall a\in A$. This means that the gains of some players are the loses of the others, and hence, zero-sum games model situations of extreme competition among players. In a zero-sum game of two players with a discrete set of actions, the payoff matrix satisfy that $R_{1}=-R_{2}=R$: player 1 maximizes the payoffs from *R* whereas player 2 tries to minimize the same payoff matrix *R*. If the sum of the payoffs is different from zero, then the game is called non-zero sum game. These games can model very different situations, ranging from extreme competition (i.e, the zero-sum case) until fully cooperative games (i.e., when all players have the same payoff function).

### 4.2. Problem Description

**Figure 2:**
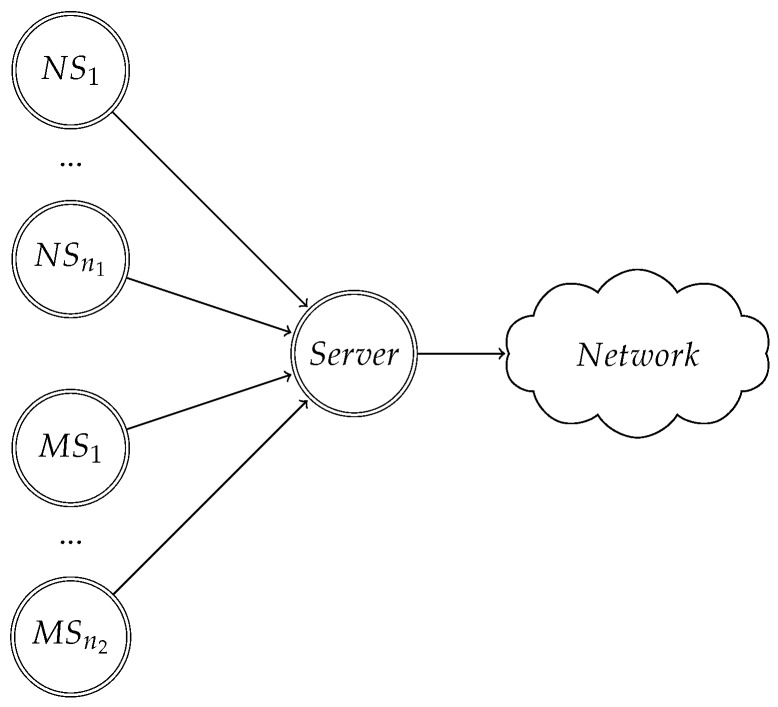
Network scheme for the case that there are $n_{1}$ normal stations (NS) and $n_{2}$ malicious stations (MS). NS respect 802.11 binary exponential backoff, whereas MS can choose to use it or to use a uniform backoff.

We use the network scheme in Figure 2 to model the CSMA/CA problem that arises when some stations modify their backoff procedure. There will be $n_{1}$ normal stations (NS), which always follow the binary exponential backoff; and $n_{2}$ malicious stations (MS), which can choose between using the binary exponential backoff or the uniform backoff. We denote by *n* the number of stations, with $n=n_{1}+n_{2}$. All *n* stations are connected to a gateway, called server, which forwards their packets to a network. The stations communicate with the server: we only consider the uplink in the problem. Observe that this problem arises in a situation in which a star topology is used. For convenience, we will denote the malicious stations as clients.

The players of the game will be the server on one side, and the clients on the other. Thus, there will be $N_{p}=n_{2}+1$ players (where $N_{p}$ denotes the number of players). Each client tries to maximize the throughput available to it, whereas the server tries to enforce that all stations in the network obtain a fair throughput. By fair, we mean that no station is getting a higher throughput at expense of others. Under the saturation condition imposed before, this means that all clients receive the same proportion of the total throughput.

The clients will have two different actions: either they behave selfishly (*s*) by using the uniform backoff or they do not behave selfishly (ns) by using the binary exponential backoff. The server will also have two actions: it can detect (*d*) if the network throughput is begin fairly distributed or not to detect (nd). If the server detects and catches a client behaving selfishly, it will drop its packet, as a punishment. This means that the client has to send again the packet, and the higher throughput advantage it had obtained by modifying its backoff vanishes. We must also take into account that this detection procedure cannot be free of charge for the server: there must be a cost associated to the detection procedure in terms of computational resources. Two of the possible schemes that could be used to detect this selfish behavior are [12], which is based in Kolmogorov-Smirnov (K-S) statistics, and [11], which is based on a modified Cramer-von Mises (C-M) test [25]. To simplify the modellinfg, we will assume that the server is able to perfectly detect when a station behaves selfishly.

### 4.3. Two Player Case: $N_{p}=2$

Now, we center in the case when $n_{2}=1$, that is, there are only two players in the game: the server and one client. We proceed to describe the payoffs for each player. We denote by $S_{1}^{ns}$ the throughput that the client can obtain by playing ns. In that case, the $n_{1}$ normal stations will obtain each a throughput $S_{n_{1}}^{ns}=S_{1}^{ns}=S^{ns}$, that is, all stations obtain the same amount of throughput (cases (a,e) in Figure 1). If the client plays *s*, it obtains a throughput $S_{c}^{1}$ if the server plays nd. This causes the normal stations to have a lower throughput, $S_{n_{1}}^{s}<S_{1}^{s}$, as observed in Figure 1.

We define $-k_{d}$ (with $k_{d}>0$) as the cost of detecting malicious behavior for the server. We model the cost function for client and server as a linear function of the throughput, with $k_{s}$ and $k_{j}$ as a constant for the server and the client respectively. Finally, we will assume that both players are maximizers, hence, they try to maximize the payoff function, that we define as follows:

- If they play (nd,s) (the first action corresponds to the server, the second to the client), the client is modifying its backoff and hence, the throughputs in the network. The server does not detect this modification, and hence, does not punish the client. Thus, the client obtains a throughput increment, which provides it a gain of $k_{1}\left(S_{1}^{s}-S^{ns}\right)$. The server has a cost proportional the throughput loss that the normal stations suffer: $k_{s}n_{1}\left(S_{n_{1}}^{s}-S^{ns}\right)$.
- If they play (d,s), the client modifies its backoff, but it is caught by the server, who drops its packet. This causes the client a loss of $k_{1}\left(0-S^{ns}\right)=-k_{1}S^{ns}$. The server has a gain proportional to the throughput that the normal stations would have lost minus the cost of the detection: $k_{s}n_{1}\left(S^{ns}-S_{n_{1}}^{s}\right)-k_{d}$.
- If they play (d,ns), the client does not modify its backoff and hence, does not affect the throughput. Hence, it has no gain nor lose. However, the server detects and hence, it incurs in the cost of detection, expressed as $-k_{d}$.
- If they play (nd,ns), the client again has no gain nor lose. The server does not detect and hence, it incurs in no cost since the client is behaving properly: it also has no gain nor lose.

All of these payoffs do not vary along the game, provided that there is no modification of the game conditions (e.g., the number of players). Since $N_{p}=2$, we can pose the game as a bimatrix, non-zero sum, static game, whose game payoffs as a function of the player actions are in Table 2.

In order to simplify, we will substitute the payoff values in Table 2 for the following constants, where $R_{1}$ is the payoff matrix for player 1 and $R_{2}$ for player 2:

(13) $$R_{1}=\left(\begin{array}{cc} -\alpha_{m} & 0\\ \alpha_{c} & -\alpha_{f} \end{array}\right)\;R_{2}=\left(\begin{array}{cc} \beta_{s} & 0\\ -\beta_{c} & 0 \end{array}\right)$$

Observe that all parameters in (13) are strictly positive, that is, $\alpha_{c},\alpha_{m},\alpha_{f},\beta_{s},\beta_{c}\in \left(0,+\infty \right)$. This arises because:

- $k_{1}$, $k_{c}$, $k_{d}$, $n_{1}$ and all the throughput values ($S_{n_{1}}^{s}$, $S_{1}^{s}$, $S^{ns}$) are all strictly positive parameters.
- The throughput of the client must be higher if it behaves selfishly than if it does not. If that were not the case, this would mean that the client achieves higher throughput by following the exponential binary backoff - and from Figure 1, we see that this is not the case if there is only one client ($n_{2}=1$). This means that $S_{1}^{s}>S^{ns}$.
- The throughput of the normal stations must decrease when the client behaves selfishly with respect to their throughput when the client follows the binary exponential backoff. As we observe in Figure 1, that is indeed the case if there are malicious stations (i.e., $n_{2}\geq 1$). This means that $S^{ns}>S_{n_{1}}^{s}$.
- It must happen that $k_{s}n_{1}\left(S^{ns}-S_{n_{1}}^{s}\right)>k_{d}$ (observe that the previous point showed that the left hand side is positive). This simply means that the cost of detecting is lower than the gain of detecting a deviation from the client. If that did not happen, it would be counterintuitive: the server incurs in a loss when it successfully detects a deviation from the client.

Observe that our model includes the case in which there is no selfishness in the client as a particular case. If the server knows that the client will always play ns (i.e., like a normal station), then the server will always play nd and hence, both players receive a payoff of 0.

### 4.4. Extension to $N_{p}>2$

The payoff functions derived in the previous section for the case that there are only two players can be extended to the general case when there are more than two players. In this case, again, there is one server which can choose between two pure actions (*d*, nd) and there will $n_{2}>1$ clients, each client being able to choose between two pure actions (*s*, ns). In the general case, the payoff function of each player will be a multidimensional array of dimensions $na_{1}\times na_{2}\times \ldots \times na_{N_{p}}$, where $na_{i}$ denotes the number of pure actions available to player $i\in \{1,N_{p}\}$. Observe that when $N_{p}=2$, each player payoff function is a matrix.

We define a vector of pure actions as $a_{p}=\left(a_{p,1},a_{p,2},\ldots ,a_{p,N_{p}}\right)$. Observe that the payoff multidimensional array contains a payoff value for each possible vector $a_{p}$. In order to obtain the payoff function of each player, for each $a_{p}$, we will define $n_{2}^{s}$ as the number of clients that play pure action *s* and $n_{2}^{ns}=n_{2}-n_{2}^{s}$ as the number of clients that play pure action ns. The payoff each player receives will be coupled with the actions of the rest of the players: in general, it will be a function $f_{i}\left(a_{p}\right)$, where *i* denotes a concrete player. There will be a payoff function for each $a_{p}$.

The payoff function for the server will depends on $a_{p}$ as follows. If the server plays *d*, the payoff function of the server will be $k_{s}n_{1}\left(S^{ns}-S_{n_{1}}^{s}\right)-k_{d}$. Remark that $S^{ns}$ is obtained considering that there are $n=n_{1}+n_{2}$ stations. Also, there will be different possible values of $n_{2}^{s}$, thus $S_{n_{1}}^{s}$ will depend on the number of clients playing *s*. Finally, observe that if all clients played ns, $n_{2}^{s}=0$ and hence, $S^{ns}=S_{n_{1}}^{s}$; thus, the payoff of the server in this case is $-k_{d}$.

If the server plays nd, the payoff value for each $a_{p}$ is $k_{s}n_{1}\left(S_{n_{1}}^{s}-S^{ns}\right)$ . It is the same as when the server played *d*, but now there is no cost $k_{d}$ and the sign is reversed.

The payoff for client $j,j\in \{1,\ldots ,n_{2}\}$ if it plays ns will be 0. If client *j* plays *s* and the server plays *d*, the payoff for client *j* will be $-k_{j}S^{ns}$. If client *j* plays *s* and the server plays nd, the payoff for client *j* will be $k_{j}\left(S_{j}^{s}-S^{ns}\right)$. Observe that $S_{j}^{s}$ will depend on $n_{2}^{s}$.

We follow this procedure for each $a_{p}$ value in order to obtain the payoff values. Observe that if $n_{2}=1$, all the expressions in this section reduce to the ones given in the previous section.

## 5. Game Theory Analysis of the CSMA/CA Problem When $N_{p}=2$

In this section, we solve analytically the CSMA/CA static game, for the case in which $N_{p}=2$, that is, for the two player case. We also provide an algorithm to solve the game for $N_{p}\geq 2$, that is, for an arbitrary number of players.

### 5.1. Nash Equilibrium Concept

The CSMA/CA game posed when $N_{p}=2$ is a non-zero sum, two player game. A very popular equilibrium concept for these games is the Nash equilibrium (NE) concept: it defines a situation in which no player can obtain a better payoff by deviating unilaterally. Non-zero sum games might have more than one NE (Chapter 3, [24] and finding all of them might be hard (see [26,27,28]). However, it is well known that every non-zero sum game has, at least, one NE in mixed strategies (Theorem 3.2, [24]). In a mixed equilibrium, each player has access to a randomizing device which outputs which pure action the player should play, with a given probability. This probability is the mixed NE.

If there are two players, each of them with two actions to choose, we can define the payoff matrices as:

(14) $$R_{1}=\left(\begin{array}{cc} r_{11}^{1} & r_{12}^{1}\\ r_{21}^{1} & r_{22}^{1} \end{array}\right)\;R_{2}=\left(\begin{array}{cc} r_{11}^{2} & r_{12}^{2}\\ r_{21}^{2} & r_{22}^{2} \end{array}\right)$$

and we can obtain a mixed NE as it is shown in (Chapter 3, [24]). We define *y* as the probability that player 1 chooses her action 1, and 1−y the probability that she chooses action 2 (for player 2, we define in an equivalent form *z* and 1−z). The equilibrium conditions are the following:

(15) $$\begin{array}{c} {\left(y_{v}^{*}\right)}^{T}R_{1}z_{v}^{*}\geq y_{v}^{T}R_{1}z_{v}^{*}\;\;{\left(y_{v}^{*}\right)}^{T}R_{2}z_{v}^{*}\geq {\left(y_{v}^{*}\right)}^{T}R_{2}z_{v} \end{array}$$

where $y_{v}=\left(y,1-y\right)$, $z_{v}=\left(z,1-z\right)$ and $y_{v}^{T}$ denotes the transposed of vector $y_{v}$. In (15) we assume that both players are maximizers: otherwise, the inequality is reversed. One mixed NE for (15) can be obtained as (pp. 85–87, [24]):

(16) $$\begin{array}{c} y^{*}=\frac{r_{22}^{2}-r_{21}^{2}}{r_{11}^{2}+r_{22}^{2}-r_{21}^{2}-r_{12}^{2}}\;\;z^{*}=\frac{r_{22}^{1}-r_{12}^{1}}{r_{11}^{1}+r_{22}^{1}-r_{21}^{1}-r_{12}^{1}} \end{array}$$

where $y^{*}\in \left[0,1\right]$ and $z^{*}\in \left[0,1\right]$ is the mixed NE.

### 5.2. Nash Equilibrium Solution to the CSMA/CA Game

The CSMA/CA game can be solved using the mixed NE concept. Using (16), the game presents the following NE, where the payoff matrices used are (13):

(17) $$\begin{array}{c} y^{*}=\frac{\beta_{c}}{\beta_{c}+\beta_{s}}\;\;z^{*}=\frac{\alpha_{f}}{\alpha_{f}+\alpha_{m}+\alpha_{c}} \end{array}$$

This means that the server plays *d* with probability $1-y^{*}$ and nd with probability $y^{*}$. The client plays *s* with probability $z^{*}$ and ns with probability $1-z^{*}$. We define the expected payoff that each player obtains if they play mixed strategies with probability (y,1−y) for the server and (z,1−z) for the client as:

(18) $$\begin{array}{cc} u_{1}\left(y,z\right)= & \left(y,1-y\right)R_{1}{\left(z,1-z\right)}^{T}=-zy\left(\alpha_{m}+\alpha_{c}+\alpha_{f}\right)+z\left(\alpha_{c}+\alpha_{f}\right)+\alpha_{f}\left(y-1\right)\\ u_{2}\left(y,z\right)= & \left(y,1-y\right)R_{2}{\left(z,1-z\right)}^{T}=zy\left(\beta_{s}+\beta_{c}\right)-z\beta_{c} \end{array}$$

Thus, the payoff that each player receives by playing their mixed NE strategy, from (17) and (18) is:

(19) $$\begin{array}{c} u_{1}=-\frac{\alpha_{f}\alpha_{m}}{\alpha_{m}+\alpha_{c}+\alpha_{f}}\;\;u_{2}=0 \end{array}$$

The values in (19) show that the equilibrium payoff for the client is 0, regardless of the game parameters. The equilibrium payoff for the server will depend on the values that the α parameters take. This means that the client can always guarantee a throughput as good as if he behaved normally. The server will have always a loss, derived from the cost of detecting ($k_{d}$, collected by the parameter $\alpha_{f}$).

### 5.3. Correlated Equilibrium Concept

Another important equilibrium concept is the correlated equilibrium (CE) concept, owed to Aumann [29], which generalizes NE concept. It assumes that there is a correlating device that produces a signal sent to both players: the players use this signal to coordinate. Each signal of the correlating device corresponds to a pure action for each player. The CE is defined so that it has no advantage to any player deviating from the prescription of the correlating device.

A CE for $N_{p}=2$ players is defined as a distribution probability ϕ(a) over the set of joint pure actions of the players $A=A_{1}\times A_{2}$, where $a=(a_{1},a_{2})$ is a vector of pure actions such that a∈A. The equilibrium condition that must be satisfied for every player i∈{1,2} is [29,30]:

(20) $$\begin{array}{c} \sum_{a_{-i}\in A_{-i}}\phi \left(a_{-i}|a_{i}\right)u_{i}\left(a_{i},a_{-i}\right)\geq \sum_{a_{-i}\in A_{-i}}\phi \left(a_{-i}|a_{i}\right)u_{i}\left(a_{i}^{\prime },a_{-i}\right)\;\forall a_{i}^{\prime }\in A_{i},\;a_{i}\neq a_{i}^{\prime } \end{array}$$

where $A_{-i}$ is the set of pure actions of the other player: $A_{-i}=A_{2}$ for i=1 and $A_{-i}=A_{1}$ for i=2.

The concept of CE is a generalization of NE concept: every NE will be a CE (but the converse is not true). Yet CE are less expensive to compute (see [31,32]). Also CE will, in general, provide different solutions to a game.

### 5.4. Correlated Equilibrium Solution to the CSMA/CA Game

The CSMA/CA game can be solved using the CE concept. The equilibrium condition (20) becomes:

(21) $$\begin{array}{cc} \sum_{a_{2}=\left\{s,ns\right\}}\phi \left(a_{2}|d\right)u_{1}\left(d,a_{2}\right) & \geq \sum_{a_{2}=\left\{s,ns\right\}}\phi \left(a_{2}|d\right)u_{1}\left(nd,a_{2}\right)\\ \sum_{a_{2}=\left\{s,ns\right\}}\phi \left(a_{2}|nd\right)u_{1}\left(nd,a_{2}\right) & \geq \sum_{a_{2}=\left\{s,ns\right\}}\phi \left(a_{2}|nd\right)u_{1}\left(d,a_{2}\right)\\ \sum_{a_{1}=\left\{d,nd\right\}}\phi \left(a_{1}|s\right)u_{2}\left(s,a_{1}\right) & \geq \sum_{a_{1}=\left\{d,nd\right\}}\phi \left(a_{1}|s\right)u_{2}\left(ns,a_{1}\right)\\ \sum_{a_{1}=\left\{d,nd\right\}}\phi \left(a_{1}|ns\right)u_{2}\left(ns,a_{1}\right) & \geq \sum_{a_{1}=\left\{d,nd\right\}}\phi \left(a_{1}|ns\right)u_{2}\left(s,a_{1}\right) \end{array}$$

Replacing the payoffs from (13), (21) becomes:

(22) $$\begin{array}{cc} \alpha_{c}\phi \left(s|d\right)-\alpha_{f}\phi \left(ns|d\right) & \geq -\alpha_{m}\phi \left(s|d\right)+0\phi \left(ns|d\right)\\ -\alpha_{m}\phi \left(s|nd\right)+0\phi \left(ns|nd\right) & \geq \alpha_{c}\phi \left(s|nd\right)-\alpha_{f}\phi \left(ns|nd\right)\\ -\beta_{c}\phi \left(d|s\right)+\beta_{s}\phi \left(nd|s\right) & \geq 0\phi (d|s)+0\phi (nd|s)\\ 0\phi (d|ns)+0\phi (nd|ns) & \geq -\beta_{c}\phi \left(d|ns\right)+\beta_{s}\phi \left(nd|ns\right) \end{array}$$

We know that the following is satisfied:

(23) $$\begin{array}{c} \phi \left(a|b\right)=\frac{\phi (a\cap b)}{\phi (b)}\;\;\phi \left(a\cap b\right)=\phi \left(b\cap a\right) \end{array}$$

We simplify (22) using (23). We use the following shorthand notation: $\phi_{11}=\phi \left(nd\cap s\right)$, $\phi_{12}=\phi \left(nd\cap ns\right)$, $\phi_{21}=\phi \left(d\cap s\right)$ and $\phi_{22}=\phi \left(d\cap ns\right)$. Observe that this is the joint distribution probability, considering that the first subscript refers to the pure action of the server, and the second, to the pure action of the client. We also consider that pure action 1 for the server is nd, and pure action 2, *d*; for the client, *s* will be its pure action 1 and ns its pure action 2. Using all this, (22) becomes:

(24) $$\begin{array}{cc} -\alpha_{m}\phi_{11}+0\phi_{12} & \geq \alpha_{c}\phi_{11}-\alpha_{f}\phi_{12}\\ \alpha_{c}\phi_{21}-\alpha_{f}\phi_{22} & \geq -\alpha_{m}\phi_{21}+0\phi_{22}\\ \beta_{s}\phi_{11}-\beta_{c}\phi_{21} & \geq 0\phi_{11}+0\phi_{21}\\ 0\phi_{12}+0\phi_{22} & \geq \beta_{s}\phi_{12}-\beta_{c}\phi_{22} \end{array}$$

where we assumed that ϕ(nd)>0, ϕ(d)>0, ϕ(s)>0 and ϕ(ns)>0. By taking into account that all α and β parameters are greater than 0 (that is, α,β∈(0,+∞)), and also restraining ϕ to be a valid distribution, we obtain the following simplified equilibrium conditions from (24):

(25) $$\begin{array}{cc} \phi_{11}\phi_{22} & =\phi_{12}\phi_{21}\\ \frac{\beta_{s}}{\beta_{c}} & =\frac{\phi_{22}}{\phi_{12}}\\ \frac{\alpha_{c}+\alpha_{m}}{\alpha_{f}} & =\frac{\phi_{22}}{\phi_{21}}\\ \phi_{11}+\phi_{12}+\phi_{21}+\phi_{22} & =1\\ \phi_{ij} & \geq 0,\;i=\{1,2\},\;j=\{1,2\}\\ \alpha ,\beta & \in (0,+\infty ) \end{array}$$

The system in (25) has only one solution:

(26) $$\begin{array}{cc} \phi_{11} & =\frac{\alpha_{f}}{\alpha_{c}+\alpha_{m}+\alpha_{c}}\frac{\beta_{c}}{\beta_{c}+\beta_{s}}\;\;\phi_{12}=\frac{\alpha_{c}+\alpha_{m}}{\alpha_{c}+\alpha_{m}+\alpha_{c}}\frac{\beta_{c}}{\beta_{c}+\beta_{s}}\\ \phi_{21} & =\frac{\alpha_{f}}{\alpha_{c}+\alpha_{m}+\alpha_{c}}\frac{\beta_{s}}{\beta_{c}+\beta_{s}}\;\;\phi_{22}=\frac{\alpha_{c}+\alpha_{m}}{\alpha_{c}+\alpha_{m}+\alpha_{c}}\frac{\beta_{s}}{\beta_{c}+\beta_{s}} \end{array}$$

Thus, there is only one CE which corresponds to the mixed NE we already found in (17): observe that $\phi_{11}=y^{*}z^{*}$, $\phi_{12}=y^{*}\left(1-z^{*}\right)$, $\phi_{21}=\left(1-y^{*}\right)z^{*}$ and $\phi_{22}=\left(1-y^{*}\right)\left(1-z^{*}\right)$. This happens with all games following the payoff matrices from (13). The payoff for each player if they follow the CE is:

(27) $$\begin{array}{c} u_{1}=-\alpha_{m}\phi_{11}+\alpha_{c}\phi_{21}-\alpha_{f}\phi_{22}\;\;u_{2}=\beta_{s}\phi_{11}-\beta_{c}\phi_{21} \end{array}$$

The payoffs obtained using CE (replacing (26) in (27)) are the same that were obtained using mixed NE, in (19). This is obvious: both are the same equilibrium.

### 5.5. Learning Algorithms: Regret Matching

There are algorithms for learning static equilibria. One of the simplest and best known is Regret Matching (RM) algorithm, proposed by Hart and Mas-Colell [18,33]. It is a simple, adaptive strategy that guarantees that the joint distribution of play converges to the set of correlated equilibria of the underlying game if each player plays a regret matching strategy [33]. The main idea of RM is to play a static game repeatedly and update a regret measure for each player depending on the outcome the players obtain each time they play the game. This algorithm requires that each player knows only her payoff and the actions of the other players (i.e., a player does not need to know the payoff functions of the other players). Each time the game is played, the regret $W_{i}\left(a_{i}^{\prime }\right)$ is obtained as:

(28) $$W_{i}\left(a_{i}^{\prime }\right)=u_{i}\left(a_{i}^{\prime },a_{-i}\right)-u_{i}\left(a_{i},a_{-i}\right),\;\forall a_{i}^{\prime }\in A_{i}$$

where $a_{i}$ is the pure action played by player *i*, $a_{-i}$ denotes the pure actions played by all the other players, $a_{i}^{\prime }$ is used to denote all pure actions available to player *i* and $A_{i}$ is the set of pure actions for player *i*. If $W_{i}\left(a_{i}^{\prime }\right)>0$, RM will assign positive probability to play $a_{i}^{\prime }$ in the future, because the player would have gained in the past if she had played $a_{i}^{\prime }$. On the other hand, if $W_{i}\left(a_{i}^{\prime }\right)\leq 0$, the player will assign probability 0 to play $a_{i}^{\prime }$. At the beginning of the game, all regrets are initialized to 0, and they are updated with each repetition of the static game following:

(29) $$W_{i}^{t+1}\left(a_{i}^{\prime }\right)=W_{i}^{t}\left(a_{i}^{\prime }\right)+W_{i}\left(a_{i}^{\prime }\right),\;\forall a_{i}^{\prime }\in A_{i}$$

where $W_{i}^{t}\left(a_{i}^{\prime }\right)$ is the regret at the beginning of the previous iteration *t* and $W_{i}\left(a_{i}^{\prime }\right)$ is obtained using (28). Observe that subscripts denote players, and superscripts denote time.

At the beginning of the static game *t*, each player chooses a pure action randomly following a distribution $p_{i}\left(a_{i}\right)$, where $p_{i}\left(a_{i}\right)$ is the probability that player *i* uses pure action $a_{i}$. $p_{i}\left(a_{i}\right)$ is obtained at the beginning of each static game as follows:

- If $W_{i}\left(a_{i}\right)\leq 0,\;\forall a_{i}\in A_{i}$, then choose a pure action randomly following the uniform distribution $p_{i}\left(a_{i}\right)$:
  (30) $$p_{i}\left(a_{i}\right)=\frac{1}{|A_{i}|}$$
  where $|A_{i}|$ stands for the number of pure actions available to player *i*.
- If there are regrets strictly higher than zero, then choose a pure action randomly following this distribution $p_{i}\left(a_{i}\right)$:
  (31) $$p_{i}\left(a_{i}\right)=\left\{\begin{array}{ccc} \frac{W_{i}^{t}\left(a_{i}\right)}{W} & \mathrm{if} & W_{i}^{k}\left(a_{i}\right)>0\\ 0 & \mathrm{if} & W_{i}^{t}\left(a_{i}\right)\leq 0 \end{array}\right.$$
  where
  (32) $$W=\sum_{a_{i}\in A_{i}|W_{i}^{t}\left(a_{i}\right)>0}W_{i}^{t}\left(a_{i}\right)$$
  that is, *W* is the sum of all positive regrets in game *t*. Observe that *W* is computed in each iteration, as the vector $W_{i}^{t}$ is updated in each iteration. This definition of *W* guarantees that $p_{i}\left(a_{i}\right)$ in (31) is an actual distribution: it sums 1 and has nonnegative components.

**Algorithm 1** Regret matching for each player.1:Initialize $W_{i}=\left(0,0,\ldots ,0\right)$, the dimension of $W_{i}$ is $|A_{i}|$2:Fix *T*, the number of iterations3:**for** t∈{1,2,…,T} **do**
4: **if** $\max\{W_{i}^{t}\}\leq 0$ **then**5:  Assign $a_{i}^{t}$ following (30)6:**else**7:  Assign $a_{i}^{t}$ following (31) and (32)8: **end if**9: Obtain payoff $u_{i}^{t}\left(a_{1}^{t},a_{2}^{t}\right)$ for i∈{1,2}10:  Update regrets using (29) and (28)11:**end for**12:**return** Strategies using (33)


We use RM to obtain the solution for the CSMA/CA game. A possible implementation for each player is found in Algorithm 1. If the static game is played *T* times, we obtain the equilibrium strategies $\hat{y}$ for player 1 and $\hat{z}$ for player 2 as:

(33) $$\begin{array}{c} \hat{y}=\frac{\sum_{t=1}^{T}a_{1}^{t}}{T},\;\hat{z}=\frac{\sum_{t=1}^{T}a_{2}^{t}}{T} \end{array}$$

where $a_{i}^{t}$ denotes the pure action taken by player *i* in time *t*. We know RM converges to the set of correlated equilibria [18], which in our game is only one point (see (26)). This correlated equilibrium is also the only Nash equilibrium of the game (see (17)). Hence, in the CSMA/CA game with two players, RM will converge to the Nash equilibrium.

## 6. Simulations for the CSMA/CA Game

We perform some simulations in order to observe and compare the theoretical developments done in previous sections. We define network using the model in Figure 2. We set the number of stations to n=5, we use BA mechanism and $T_{p,l}$ (long payload) in order to estimate the network throughput using Bianchi’s model. The parameters of normal stations, denoted by subscript 1 will be $W_{1}=32$, $CW_{max,1}=1024$, and hence, $m_{1}=5$ (extracted from IEEE 802.11 standard). The malicious stations, denoted with subscript 2, will use the uniform random mechanism modification described in Section 3, with a window length $W_{2}=8$. The rest of IEEE 802.11 parameters are in Table 1, taken from [22]. We solve equations (3) to (10), and obtain the throughput values for different number of malicious stations: $n_{2}\in \left\{1,2,3,4\right\}$.

We also need to define the parameters that are used to model the payoff functions. We use $k_{s}=k_{c}=1$, $k_{d}=0.1$. The payoff functions are obtained using Table 2 for the case of two players and the procedure in Section 4.4 for the case $N_{p}>2$. For two players, $S^{ns}=0.1617$, $S_{n}^{s}=0.0700$, $S_{c}^{s}=0.5225$, which gives rise to the payoff matrix in Table 3.

### Simulation 2: Myopic Solutions

We can use (17) and Table 3 to obtain the theoretical solutions for the two player game. The mixed equilibrium actions are $y_{n}=0.3095$ and $z_{n}=0.1364$, which yield a payoff of −0.05 for the server and 0 for the client. Recall that the CE, obtained using (26), yields the same equilibrium.

**Figure 3:**
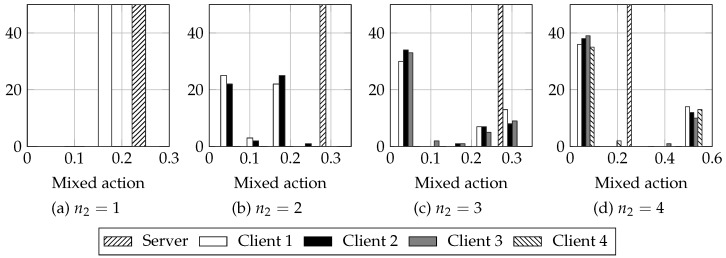
Histogram of actions obtained using RM algorithm, for n=5 stations and variable number of malicious stations. Each histogram is computed using 5 bins. Cl stands for client. Observe that the action of the server does not vary significantly, whereas the actions of the clients do. Also, observe how as $n_{2}$ increses, the clients histogram presents two peaks: the biggest close to 0 and a smaller peak at another mixed action value. This hints that the game tends to the two player case when there are many clients: all but one client tend to behave as normal stations.

Then, we simulate using RM algorithm for $n_{2}\in \left\{1,2,3,4\right\}$. We set the number of iterations T=2000, and run the learning process 50 times. The empirical payoffs obtained are in Table 4, and in Figure 3, the histogram of the mixed actions obtained is represented for all the $n_{2}$ cases tested. We can compare to the theoretical results expected in the two player case, by computing the difference between the actions and payoff obtained using RM (Algorithm 1) and the theoretical values using (17) and (19). The mean difference in mixed actions is −0.0224±0.0183 (mean ± standard deviation) for the server and 0.0056±0.0087 for the client. The mean difference in payoffs is also small: 0.0007±0.0024 for the server and −0.0015±0.0013 for the client. Thus, RM provides a very good approximation to the expected game values.

**Figure 4:**
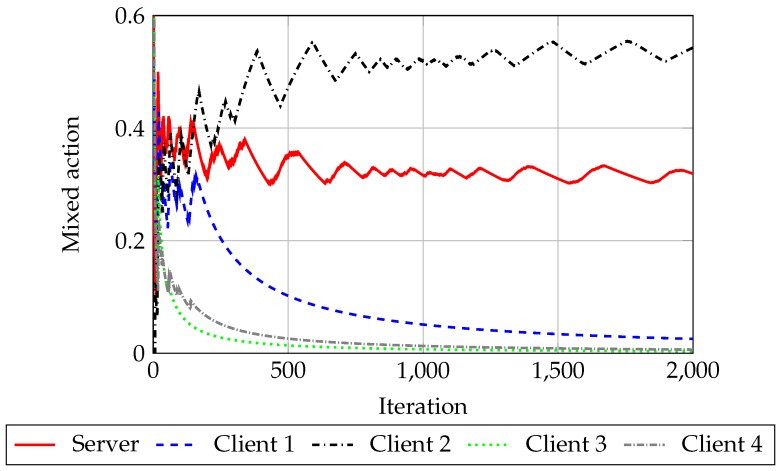
Example of the evolution of the mixed action for each player, using RM algorithm, where Cl stands for client. In each simulation, all clients tend to play ns, except for one. This one randomly arises at each simulation using RM algorithm. This means that the game tends to the two player situation.

It is of special interest noting that, for $n_{2}\geq 2$, each of the clients distribution presents two peaks, clearer as $n_{2}$ grows; one of them is nearly 0. We observe that in each game realization all clients but one tend to behave as normal stations (i.e., they tend to play ns), as can be observed also in Figure 4 for $n_{2}=4$: client 1 plays a mixed action around z=0.5 and the rest of clients tend to play z=0, that is, they tend to always play ns. This means that the game tends to the two player case, even if there are more than two players. This might be due to having payoffs such that they do not encourage having more than one player behaving selfishly at once. As we saw in Figure 1, as the number of clients increased, the advantages of playing *s* for the clients decreased: the difference between the normal behavior throughput and the throughput obtained when using a different backoff shrank. Since the payoff of the clients is proportional to this difference, it is not enough gain for them to play ns: the loss when they play *s* and the server plays *d* do not compensate the gains when they play *s* and the server plays nd; hence, it is better for them playing ns.

## 7. Conclusions

In this paper, we study a CSMA/CA wireless sensor network under a backoff attack: some of the sensors of the network are malicious and deviate from the defined contention mechanism. We first use Bianchi’s network model to theoretically study the network throughput and observe that the malicious sensors have a gain on throughputs, at the expense of other sensors in the network. Even though the total throughput in some situations stays the same, it is not fairly distributed among sensors. This effect depends on the backoff parameters used by the malicious sensors, as well as on the number of malicious sensors present in the network.

We then proceed to model the situation as a static game between the malicious sensors and a network gateway (thus, using a star topology): the gateway (server) tries to enforce the malicious sensors to behave following the contention mechanism, whereas the malicious sensors try to obtain a higher throughput. We solve analytically the game for the case that there is only one malicious sensor and propose an algorithm based on Regret-Matching to learn the equilibrium with any number of players. Our approach is validated via simulations.

The framework we introduce in this paper can be further deepened in different ways. The malicious sensors could vary their parameters (for instance, their contention window) in order not to be easily detected by the defense system: this would mean that the action set of the client grows up and also, the game complexity. Another line of research would be modeling the game using dynamic game tools: in this case, the stations would choose their actions in order to maximize not their immediate rewards, but taking into account future interactions: in a wireless network, it is rare that the stations communicate only once.

Finally, our approach shows that there is a trade-off between modeling complexity and computational complexity. By making use of payoff matrices, we alleviate this trade-off: the game theoretic solutions we provide are agnostic with regards to where these payoffs come from. That is, we could use Bianchi’s model as we do to relate rewards with the throughput, or we could relate rewards to other network parameters (as delay or any other measure of the quality of service) and yet our game modeling would be valid: we should only replace the payoff matrix and solve the game, with these new matrices. Hence, we believe that we introduce a framework simple enough to accommodate different situations, but also complex enough as to model the conflict and the actions of the different stations involved by using game theory tools.

## Figures and Tables

**Table 1:** Values used for simulation 1.

Parameter	Value	Parameter	Value
$T_{p,s}$	256 bits	$T_{p,l}$	8184 bits
MAC header	272 bits	PHY header	128 bits
ACK	112 bits + PHY header	RTS	160 bits + PHY header
CTS	272 bits + PHY header	Bit rate	1 Mbps
δ	1 μs	$T_{s}$	50 μs
SIFS	28 μs	DIFS	128 μs

**Table 2:** Payoffs values for the game posed, when $n_{2}=1$. The payoff vectors are of the form $u=(u_{1},u_{2})$, where $u_{1}$ is the payoff of the server and $u_{2}$ is the payoff of the client.

	s	ns
nd	$\left(k_{s}n_{1}\left(S_{n_{1}}^{s}-S^{ns}\right),k_{1}\left(S_{1}^{s}-S^{ns}\right)\right)$	$\left(0,0\right)$
*d*	$\left(k_{s}n_{1}\left(S^{ns}-S_{n_{1}}^{s}\right)-k_{d},-k_{1}S^{ns}\right)$	$\left(-k_{d},0\right)$

**Table 3:** Payoffs values for the game when $n_{1}=4$ and $n_{2}=1$. The first entry of the payoff vector is the server payoff, the second is the client payoff.

	s	ns
nd	$\left(-0.3668,0.3608\right)$	$\left(0,0\right)$
*d*	$\left(0.2668,-0.1617\right)$	$\left(-0.1,0\right)$

**Table 4:** Empirical payoffs obtained using RM for each value of $n_{2}$. The payoff vector *u* has the server payoff first and then, the payoff of each client. Observe that payoffs do not significantly vary as the number of players increase. This is consistent with Figure 4: the game tends to the two player situation, even if there are more players.

$n_{2}$	*u*
1	(−0.0493,−0.0015)
2	(−0.0504,−0.0011,−0.0012)
3	(−0.0502,−0.0011,−0.0011,−0.0013)
4	(−0.0499,−0.0008,−0.0008,−0.0004,−0.0003)

## Abbreviations

The following abbreviations are used in this manuscript:

ACK	Acknowledgement frame
BA	Basic Access mechanism
CE	Correlated Equilibrium
CS	Carrier Sense mechanism
CSMA/CA	Carrier-Sense Medium Access with Collision Avoidance
CW	Contention Window size
DCF	Distributed Coordination Function
MAC	Medium Access Control
MS	Malicious (greedy) Station
NE	Nash Equilibrium
NS	Normal Station
RM	Regret-Matching algorithm
RTS/CTS	Request-to-Send/Clear-to-Send mechanism
STA	Station
WLAN	Wireless Local Area Network
